# Identification of Terpenoid Chemotypes Among High (−)-*trans*-Δ^9^- Tetrahydrocannabinol-Producing *Cannabis sativa* L. Cultivars

**DOI:** 10.1089/can.2016.0040

**Published:** 2017-03-01

**Authors:** Justin T. Fischedick

**Affiliations:** Excelsior Analytical Laboratory, Inc., Union City, California.

**Keywords:** chemotype, hierarchical clustering analysis, orthogonal partial least squares discriminant analysis, partial least squares discriminant analysis, principal component analysis, terpenes

## Abstract

**Introduction:** With laws changing around the world regarding the legal status of *Cannabis sativa* (cannabis) it is important to develop objective classification systems that help explain the chemical variation found among various cultivars. Currently cannabis cultivars are named using obscure and inconsistent nomenclature. Terpenoids, responsible for the aroma of cannabis, are a useful group of compounds for distinguishing cannabis cultivars with similar cannabinoid content.

**Methods:** In this study we analyzed terpenoid content of cannabis samples obtained from a single medical cannabis dispensary in California over the course of a year. Terpenoids were quantified by gas chromatography with flame ionization detection and peak identification was confirmed with gas chromatography mass spectrometry. Quantitative data from 16 major terpenoids were analyzed using hierarchical clustering analysis (HCA), principal component analysis (PCA), partial least squares discriminant analysis (PLS-DA), and orthogonal partial least squares discriminant analysis (OPLS-DA).

**Results:** A total of 233 samples representing 30 cultivars were used to develop a classification scheme based on quantitative data, HCA, PCA, and OPLS-DA. Initially cultivars were divided into five major groups, which were subdivided into 13 classes based on differences in terpenoid profile. Different classification models were compared with PLS-DA and found to perform best when many representative samples of a particular class were included.

**Conclusion:** A hierarchy of terpenoid chemotypes was observed in the data set. Some cultivars fit into distinct chemotypes, whereas others seemed to represent a continuum of chemotypes. This study has demonstrated an approach to classifying cannabis cultivars based on terpenoid profile.

## Introduction

*Cannabis sativa* L. (cannabis) is an annual diecious member of the Cannabaceae family. Since ancient times cannabis has been used by humans for its fiber, seed, as well as its psychoactive and medicinal resin.^1,2^ Despite a long history of use, the legal status of cannabis in modern times often depends on its intended use. Cannabis grown for its fiber or seed, commonly known as hemp, is legally cultivated in many nations. Cannabis used for its psychoactive properties, in North American commonly known as “marijuana,” has been illegal in most nations worldwide since the 1961 United Nations Single Convention on Narcotic Drugs.^3^ Recently however, laws concerning the legal status of cannabis are changing around the world. In the United States of America, many states have legalized cannabis for medical use, whereas some have even legalized cannabis for adult consumption.^4^ Uruguay recently legalized cannabis and laws in various countries within the European Union (EU) are also changing regarding cannabis.^5,6^ Due to its many and controversial uses, the taxonomic classification of cannabis has been the subject of both legal and scientific debate.

From a morphological perspective, three main types of cannabis have been described sativa, indica, and ruderalis. Generally sativa plants are described as taller and loosely branched, whereas indica is typically shorter, more densely branched, and conical in shape. Ruderalis is described as short (≤2 feet) at maturity and sparsely if at all branched.^7^ Whether the genus *Cannabis* is monotypic and composed of just a single species (*C. sativa*) or polytypic and composed of multiple species is an old taxonomic debate.^8,9^ A more recent taxonomic classification dividing cannabis into seven putative taxa based on morphological, geographical, and genetic traits has been proposed.^1,10^

Cannabinoids are a group of terpenophenolic compounds found in cannabis. Today over 100 cannabinoids from cannabis have been characterized.^11–14^ (−)-*Trans-*Δ^9^-tetrahydrocannabinol (THC) is considered the primary active ingredient responsible for the intoxicating and medical effects attributed to cannabis. THC has antiemetic, neuroprotectant, and anti-inflammatory properties as well as the ability to reduce certain forms of neuropathic and chronic pain.^15–17^ Another important cannabinoid, cannabidiol (CBD), has neuroprotective, anti-inflammatory, antipsychotic, and antiseizure properties without the intoxicating effects of THC.^18–20^ Other minor cannabinoids, such as cannabigerol (CBG), cannabichromene (CBC), and tetrahydrocannabivarin (THCV), also exhibit interesting pharmacological properties.^17,21^

Since cannabinoids are the major active ingredients found in cannabis, it makes sense to classify cannabis from a chemotaxonomic perspective according to cannabinoid levels for both medical and legal purposes. Early studies noted that cannabis used for fiber tended to have higher levels of CBD, whereas cannabis used for drug purposes had higher levels of THC.^22^ Small and Beckstead identified three chemical types (chemotypes) based on ratios of THC and CBD: type I, which contained high THC (>0.3%) and low CBD (<0.5%), type II high THC (>0.3%) and high CBD (>0.5%), and type III high CBD (>0.5%) and low THC (<0.3%).^23^ The three chemotype concepts were confirmed by Hillig and Mahlberg among cultivars originating from different geographic locations in addition to noting other minor cannabinoids that were characteristic of certain cultivars.^24^ Studies on the inheritance of cannabinoid phenotypes have demonstrated that chemotype can be independent from the plants morphology.^25^ In recent decades drug type I cultivars have increased in potency containing upward of about 15–20% THC,^26,27^ as have type II and type III cultivars.^28–30^ Clinical research has demonstrated that the combination of THC and CBD can alter their effects^31–33^ indicating the importance of knowing active compound ratios when using cannabis for medical purposes.

Terpenoids represent another interesting group of biologically active compounds found in cannabis. Due to their volatile nature, the mono- and sesquiterpenoids found in cannabis contribute to the plants' aroma and flavor. About 100 terpenoids have been identified in cannabis, many of which are found in other plants.^11,34^ Both cannabinoids and terpenoids are produced in the trichomes of cannabis, which are found at highest density on female flower buds.^35–37^ Terpenoids are usually present in cannabis flower buds in the 0.5–3.5% range^28^ and are found at significant levels in cannabis smoke and vapor.^38^ As biologically active compounds, terpenoids may play a role in the overall effects of herbal cannabis.^17^ The popularly understood distinctions between indica and sativa may have more to do with aroma and subjective effects than plant morphology. Recent studies have shown that terpenoids are useful in distinguishing cannabis cultivars that have similar cannabinoid content.^28,39^ A study of cannabinoid and terpenoid profiles among medical cannabis samples analyzed by a cannabis testing laboratory in California found a continuum of terpenoid profiles among the wide variety of sample names.^29^ Another study found that cannabis samples described as indica contained more myrcene and hydroxylated terpenoids, whereas those described as sativa tended to contain more terpinolene, 3-carene, and a few specified sesquiterpenes.^30^

However, in the aforementioned studies, it was difficult to define specific terpenoid chemotypes (or “chemovars” as described by Hazekamp)^30,39^ associated with commonly used cultivar names. This was likely due to the wide degree of quantitative variation in the sample sets as well as the lack of any formally agreed-upon nomenclature for cannabis cultivars. Confusing and obscure nomenclature makes it difficult for doctors and patients to decide which cultivars they should use for various medical conditions. Furthermore, given recent advances in cannabis legalization, describing this chemical variation more systematically has never been more pertinent from both an agricultural and industrial perspective. Therefore, the purpose of this study was to assess the variation of terpenoid chemotypes among high THC-producing cannabis cultivars available to medical cannabis patients in the state of California. We chose to analyze a sample set obtained by monitoring the terpenoid content of samples submitted by a single medical cannabis dispensary over the course of a year. The single source was chosen with the assumption that some pattern or consistency in nomenclature would be used by the dispensary most likely based on smell or some knowledge of the source plant material.

## Materials and Methods

### Chemicals

Individual terpenoid reference standards, α-pinene, β-pinene, limonene, ocimene (mixture of isomers), α-phellandrene, terpinolene, geraniol, and α-bisabolol, were purchased from Sigma-Aldrich (St. Louis, MO); myrcene, 3-carene, and guaiol from Fluka Sigma–Aldrich; β-caryophyllene, α-humulene, caryophyllene-oxide, and β-eudesmol were purchased from Santa Cruz Biotech (Santa Cruz, CA). Two terpenoid mixes, Can-Terp Mix1 and Can-Terp Mix2, were purchased from SPEX CertiPrep (Metuchen, NJ) and contained camphor, β-myrcene, farnesene (mixture of isomers), p-mentha-1,5-diene, eucalyptol, isoborneol, linalool, β-caryophyllene, ocimene (mixture of isomers), caryophyllene oxide, fenchone, hexahydrothymol, α-bisabolol, camphene, 3-carene, cedrol, geranyl-acetate, isopulegol, nerol, *cis*-nerolidol, valencene, β-pinene, limonene, α-pinene, fenchone, borneol, geraniol, pulegone, α-humulene, α-cedrene, terpinolene, γ-terpinene, α-terpinene, guaiol, sabinene, camphor, endo-fenchyl-alcohol, *trans*-nerolidol, sabinene hydrate, and terpineol (mixture of isomers) in methanol. Methanol (MeOH) used in sample preparation was of ACS grade from Fisher Scientific (Waltham, MA).

### Sampling and sample preparation

All plant materials used in this study were submitted from a single permitted medical cannabis dispensary named the Garden of Eden in Hayward, California to an analytical testing laboratory, Excelsior Analytical Laboratory, Inc., for cannabinoid and terpenoid analysis. Samples were submitted in 2–3.5 g portions over the course of 1 year from February 2015 to February 2016, at a rate of ∼10–20 samples every couple of weeks. Samples were typically analyzed within 1–3 days of submission. Weight loss upon drying was determined on a 0.5–1 g portion of each sample in a MB 35 OHAUS moisture analyzer (OHAUS Corporation, Parsippany, NJ) according to manufacturer's specifications. Cultivar names assigned to each sample by the client were recorded. Samples (1 g±10 mg) were prepared according to previously validated methodology with the exception that methanol was used for extraction instead of ethanol.^28,39^ In brief, for quantitative analysis, samples were extracted in a 50-mL plastic tube three times with 40, 30, and 20 mL of MeOH. For each round of extraction, tubes were shaken on an orbital shaker at 200 rpm for 15 min each, after which the supernatant was transferred to a 100-mL volumetric flask. After extraction the volume was brought up to 100 mL with MeOH, and samples were filtered over a 0.22 μM PTFE membrane before gas chromatography (GC) analysis. Representative samples for peak confirmation by gas chromatography–mass spectrometry (GC-MS) were prepared by extracting 1 g (±10 mg) of flower samples with 40 mL of MeOH in a plastic tube with 30 min of shaking at 200 rpm and filtration through 0.22 μM PTFE membrane.

### Gas chromatography flame ionization detection

Terpenoids were analyzed on an Agilent Technologies (Santa Clara, CA) 6890 gas chromatograph equipped with a flame ionization detector (FID), HP 6890 injector, and autosampler. The analytical conditions were the same as previously described.^28^ In brief, the column was a Restek (Bellefonte, PA) Rtx-5 (5% diphenyl polysiloxane) 30 m, 0.25 mm ID, 0.25 μM film thickness. Nitrogen was used as a carrier gas at a flow rate of 1.2 mL/min. The injector temperature was set to 230°C, the FID at 250°C, and the oven initially at 60°C with ramp to 240°C at a rate of 3°C/min with a 5-min hold at 240°C. Injections were 4 μL with a 1:20 split ratio. The instrument was controlled by Agilent Technologies GC ChemStation software Rev B.04.03. Monoterpenoids were quantified with average response of four point calibration curves of α-pinene, β-pinene, and limonene in the range of 10 to 500 μg/mL in MeOH. Sesquiterpenoids were quantified based on four-point calibration curves (10–500 μg/mL in MeOH) of β-caryophyllene.

### Gas chromatography–mass spectrometry

Terpenoid peak identity was confirmed by GC-MS on a PerkinElmer (Waltham, MA) Clarus 680 gas chromatograph, equipped with a Clarus SQ 8T single quadrupole mass spectrometer. The GC was equipped with PerkinElmer Elite 5 (5% diphenyl polysiloxane) 30 m, 0.25 mm ID, 0.25 μM film thickness column. Hydrogen was used as a carrier gas at a flow rate of 1.5 mL/min. The injector temperature was set to 230°C, and the oven initially at 60°C with ramp to 240°C at a rate of 3°C/min. Injections were 1 μL with a 1:10 split ratio. The mass spectrometer transfer line and source temperatures were 150°C. The mass spectrometer operated with a 3 min solvent delay, after which the instrument scanned from 50 to 500 amu in 1 sec with a 0.05 interscan delay in electron impact positive mode at 70 eV. The instrument was controlled by Turbomass software Version 6.1.0.1963 (PerkinElmer). Compounds were identified based on comparison of retention times with reference standards and GC-MS confirmation.

### Data analysis

TIBCO Spotfire version 7.0.1 (TIBCO Spotfire, Boston, MA) was used to perform hierarchical clustering analysis (HCA). Wards method was used for HCA with averaged values of each terpenoid in the cultivars used for ordering weight. Principal component analysis (PCA), partial least squares discriminant analysis (PLS-DA), and orthogonal partial least squares discriminant analysis (OPLS-DA) were performed with Metaboanalyst 3.0.^40^ No scaling or centering was applied to the data before PCA, PLS-DA, or OPLS-DA analysis due to the similar concentration ranges of analytes.

## Results and Discussion

All cultivars analyzed in this study were high THC-containing varieties (>10%) as determined by high performance liquid chromatography (HPLC) (data not shown). Cultivar names, which were analyzed at least five times (*n*≥5) in our laboratory over the data collection period, were selected to develop the classification scheme. This resulted in a data set containing 233 samples with 30 different cultivar names. The weight loss upon drying was between 6% and 14% indicating that the samples were not excessively dry and had similar moisture content. Typically, samples were submitted for testing a few days before they were made available to patients and usually represented samples from different batches purchased by the dispensary from various producers (personal communication with dispensary owners). Cannabinol (CBN), the primary degradation product of THC was <0.1% in all samples. The carboxylic acid form of THC, THC-acid, which is biosynthesized by the plant and converted into THC by decarboxylation,^17,36^ dominated in all samples. Although there was no way of determining the age or exact storage conditions of the plant material, taken together, these observations suggest that the plant material was dried and stored properly.

As mentioned by Hazekamp, certain terpenoids in cannabis, mainly certain sesquiterpenoids, are difficult to identify due to poor resolution and lack of reference materials.^30^ For the purposes of classification, we chose to build a database containing only unequivocally identified terpenoids for which we had an authentic reference standard whose peak identification was confirmed by GC-MS (Table 1). It is worth noting that previous studies identified *cis*-ocimene at a relative retention time (RRT) of 0.40.^28,30,39^ In this study, we were able to reidentify this peak as *trans*-ocimene due to the availability of a reference standard that contained a known mixture of *cis*-ocimene and *trans*-ocimene isomers. Isomers could be distinguished based on known elution order^41^ and mass spectra (Supplementary Fig. S1). The quantitative levels of the 16 terpenoids in the different cultivars are shown in Table 2.

**Table 1.** **Terpenoids Relative Retention Time Compared with β-Caryophyllene**

Compound	RRT
α-Pinene	0.259
β-Pinene	0.310
Myrcene	0.324
α-Phellandrene	0.344
3-Carene	0.352
α-Terpinene	0.360
Limonene	0.377
*trans*-Ocimene	0.402
Terpinolene	0.466
Linalool	0.479
Endo-fenchyl-alcohol	0.504
α-Terpineol	0.627
Geranyl-acetate	0.940
β-Caryophyllene	1.000
Humulene	1.053
α-Bisabolol	1.390

RRT, relative retention time.

**Table 2.** **Quantitative Terpenoid Data (mg/g) in Each Cultivar**

Cultivar	Sample (*n*=#)	α-Pinene	β-Pinene	Myrcene	α-Phellandrene	3-Carene	α-Terpinene	Limonene^a^	trans-Ocimene	Terpinolene	Linalool	Endo-fenchyl-alcohol	α-Terpineol	Geranyl-acetate	β-Caryophyllene	Humulene	α-Bisabolol
Master Kush	5	<LOQ	0.6±0.4	1.3±0.3	ND	ND	ND	3.2±1.3	ND	ND	<LOQ	<LOQ	<LOQ	ND	2.7±0.5	1.1±0.2	1.1±0.2
Bubba Kush	7	ND	0.3±0.3	1.7±0.8	ND	ND	ND	3.0±1.1	ND	ND	0.7±0.4	ND	ND	ND	4.1±1.9	1.8±1.1	0.9±0.5
Mr. Nice	6	<LOQ	0.4±0.3	3.6±0.8	ND	ND	ND	3.4±1.2	0.4±0.3	ND	<LOQ	ND	ND	ND	3.0±1.4	0.8±0.5	0.5±0.3
Sour Diesel	10	<LOQ	0.6±0.3	2.8±1.1	ND	ND	ND	4.4±1.1	ND	ND	1.1±0.3	<LOQ	ND	ND	4.0±1.3	1.6±0.5	0.9±0.3
Blue Cookies	6	ND	0.4±0.3	1.0±0.3	ND	ND	ND	2.5±0.9	ND	ND	0.9±0.3	<LOQ	ND	ND	6.0±0.6	2.8±0.3	<LOQ
Girl Scout Cookies	16	ND	0.5±0.2	1.8±0.5	ND	ND	ND	3.4±1.0	ND	ND	1.5±0.6	<LOQ	<LOQ	ND	6.7±1.3	3.2±0.6	<LOQ
Animal Cookies	14	<LOQ	0.6±0.3	2.1±0.6	ND	ND	ND	3.8±0.9	ND	ND	1.6±0.4	<LOQ	<LOQ	ND	6.8±1.0	3.2±0.7	0.4±0.3
Thin Mints	6	ND	0.6±0.1	2.2±1.1	ND	ND	ND	3.3±0.9	ND	ND	1.5±0.7	ND	ND	ND	7.1±2.5	3.4±1.1	0.4±0.2
Fortune Cookies	5	ND	0.6±0.1	1.6±0.5	ND	ND	ND	3.6±1.0	ND	ND	1.8±0.4	<LOQ	0.1±0.2	ND	8.2±1.9	4.0±0.9	0.6±0.2
Sherbert	7	0.6±0.3	1.1±0.2	1.5±0.6	ND	ND	ND	5.2±0.9	<LOQ	ND	2.0±0.6	0.6±0.3	0.5±0.4	<LOQ	6.4±2.0	2.7±0.8	ND
Chemdog	6	<LOQ	0.8±0.2	3.0±3.2	ND	ND	ND	4.2±1.1	ND	<LOQ	0.9±0.2	0.5±0.3	ND	<LOQ	6.4±1.8	2.2±0.6	1.0±0.3
Gorilla Glue #4	10	<LOQ	0.6±0.3	3.8±1.3	ND	ND	ND	4.2±1.1	ND	ND	0.9±0.3	<LOQ	<LOQ	ND	7.8±1.5	2.5±0.5	0.9±0.2
Crown Og	5	0.3±0.3	1.0±0.2	7.6±5.7	ND	ND	ND	5.1±1.6	ND	ND	1.7±0.3	0.6±0.1	0.6±0.1	ND	2.7±0.8	1.1±0.3	<LOQ
Skywalker Og Kush	6	0.4±0.3	1.0±0.2	6.7±2.9	ND	ND	ND	5.1±1.5	ND	ND	2.0±0.6	0.6±0.3	0.7±0.4	ND	3.7±0.9	1.4±0.3	<LOQ
Og Kush	10	0.4±0.4	0.9±0.4	7.1±2.5	ND	ND	ND	5.4±2.5	ND	ND	1.8±1.0	0.5±0.4	0.5±0.4	ND	4.0±1.1	1.50±0.4	0.5±0.2
Superman Og Kush	7	0.5±0.3	1.2±0.3	6.4±1.6	ND	ND	ND	6.3±1.7	ND	ND	1.8±0.3	0.6±0.1	0.7±0.1	ND	3.5±0.5	1.3±0.2	<LOQ
Gas	6	0.5±0.3	1.1±0.3	6.0±1.3	ND	ND	ND	5.9±1.7	ND	ND	2.0±0.3	0.6±0.1	0.6±0.1	ND	4.0±0.6	1.5±0.2	0.4±0.2
Tahoe Og Kush	5	0.5±0.3	1.2±0.2	8.8±4.6	ND	ND	ND	6.8±1.3	ND	ND	2.1±0.6	0.6±0.1	0.6±0.1	ND	4.6±0.8	1.8±0.3	0.5±0.1
Triple O	7	0.5±0.2	1.0±0.2	3.0±5.5	ND	ND	ND	6.7±1.4	ND	ND	2.2±0.4	0.7±0.2	0.6±0.1	ND	3.5±0.5	1.4±0.2	0.4±0.3
Gelato	5	1.0±0.1	1.6±0.2	1.8±0.6	ND	ND	ND	9.1±1.6	0.1±0.2	ND	3.1±0.9	1.0±0.1	0.9±0.1	0.7±0.4	2.1±1.4	1.1±0.3	<LOQ
Miami White Kush	6	0.8±0.4	1.4±0.4	3.5±1.7	ND	ND	ND	8.3±1.3	<LOQ	ND	1.7±0.6	0.8±0.3	0.6±0.4	0.4±0.3	6.8±0.9	2.0±0.3	0.7±0.2
Jack Herer	9	<LOQ	0.7±0.3	0.9±0.6	<LOQ	ND	ND	0.9±0.4	1.1±0.7	8.3±3.0	<LOQ	ND	<LOQ	ND	2.6±0.7	1.4±0.4	ND
Trainwreck	7	0.9±0.2	1.5±0.3	3.7±2.3	0.4±0.3	<LOQ	<LOQ	3.4±1.9	2.6±1.7	9.6±4.8	0.4±0.4	<LOQ	0.5±0.3	ND	1.5±0.4	0.5±0.3	ND
Purple Cream	9	2.1±0.4	0.5±0.2	7.1±1.4	ND	ND	ND	<LOQ	1.2±0.3	ND	1.0±0.2	ND	ND	ND	3.7±0.9	1.0±0.2	<LOQ
Grape Ape	8	2.0±1.0	0.5±0.3	7.3±3.7	ND	ND	ND	<LOQ	1.2±0.6	ND	1.1±0.4	ND	ND	ND	3.5±0.5	1.0±0.1	<LOQ
Purple Princess	9	2.7±0.6	0.7±0.1	8.9±1.5	ND	ND	ND	<LOQ	1.7±0.5	ND	1.2±0.2	ND	ND	ND	4.1±1.2	1.1±0.3	<LOQ
Blue Dream	19	4.2±1.3	2.0±0.5	7.5±2.8	ND	ND	ND	1.1±0.8	ND	ND	0.7±0.3	ND	ND	ND	2.3±0.6	1.4±0.3	<LOQ
Strawberry Haze	5	1.1±0.1	0.8±0.1	7.5±1.4	ND	ND	ND	2.4±0.5	3.8±0.4	ND	1.2±0.1	ND	ND	ND	2.7±0.5	1.2±0.3	0.6±0.1
Godfather	7	2.9±1.0	1.0±0.3	12.0±4.0	ND	ND	ND	1.9±0.6	1.3±0.6	ND	0.9±0.4	ND	ND	<LOQ	1.6±0.3	0.5±0.2	ND
Purple Urkle	5	3.2±0.6	0.9±0.1	11.4±3.1	ND	ND	ND	<LOQ	1.7±0.4	ND	1.4±0.1	ND	ND	ND	3.5±0.5	1.0±0.2	<LOQ

^a^ Limonene peak overlaps with β-phellandrene mainly in cultivars containing α-phellandrene.

Plus or minus values indicate standard deviation.

<LOQ, less than limit of quantification (<0.4 mg/g); ND, not detected.

**FIG. 1.**
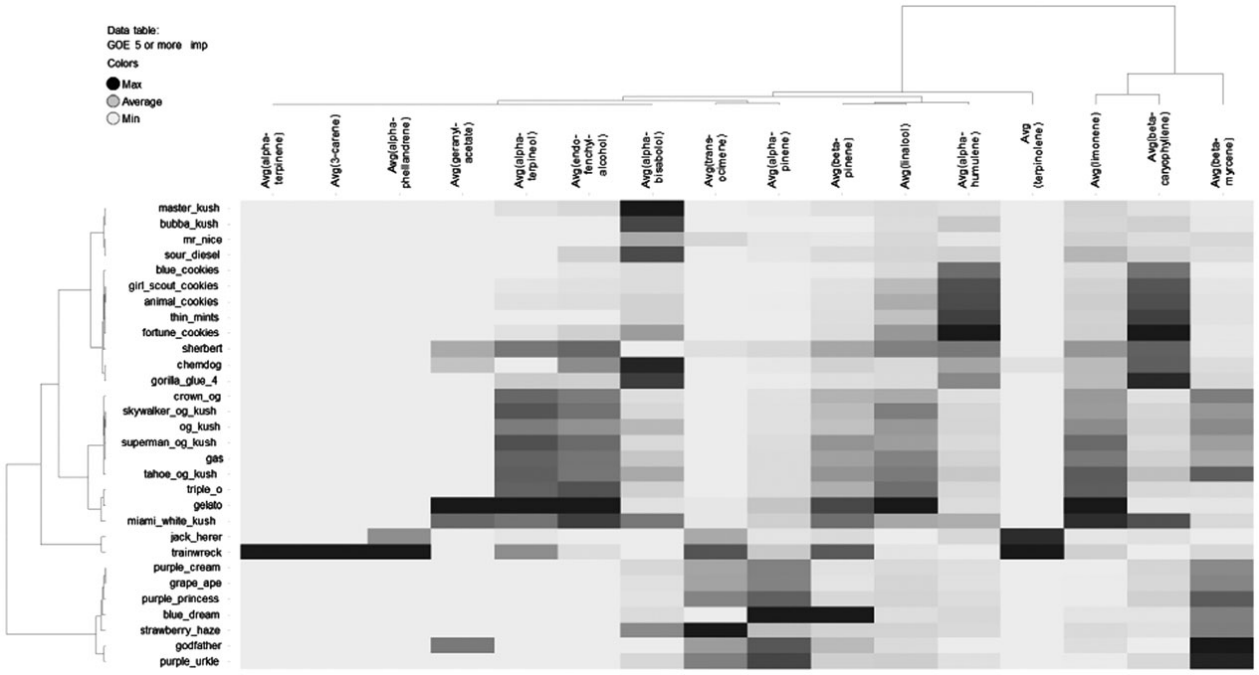
Dendrogram with heatmap generated from HCA showing average terpenoid content among the clustered cultivars. HCA, hierarchical clustering analysis.

Initially each cultivar's quantitative terpenoid data was analyzed by HCA (Fig. 1). Based on the HCA, the cultivar names could be broken down into five groups. First, a myrcene-dominant group made up of the Purple Cream, Grape Ape, Purple Princess, Blue Dream, Strawberry Haze, Godfather, and Purple Urkle cultivars. A second terpinolene-dominant group composed of the Jack Herer and Trainwreck Cultivars. Another third group composed of the cultivars named as Crown Og, Skywalker Og Kush, Og Kush, Gas, Tahoe Og Kush, Triple O, Gelato, and Miami White Kush dominated in myrcene and limonene. Distinguishing characteristics among these cultivars were the relatively higher levels of the monoterpenoid alcohols α-terpineol, endo-fenchyl-alcohol, and linalool. A fourth group of cultivars were dominated by β-caryophyllene, which included Blue Cookies, Girl Scout Cookies, Animal Cookies, Thin Mints, Fortune Cookies, Sherbert, Chemdog, and Gorilla Glue #4 (gorilla glue). A fifth group composed of Master Kush, Bubba Kush, Mr. Nice, and Sour Diesel tended to dominate in myrcene, limonene, or β-caryophyllene. One distinguishing characteristic of this group was the relatively higher levels of α-bisabolol in Master Kush, Bubba Kush, and Sour Diesel. We termed the five groups myrcene (first), terpinolene (second), Og Kush (third), caryophyllene (fourth), and bisabolol (fifth), respectively.

**FIG. 2.**
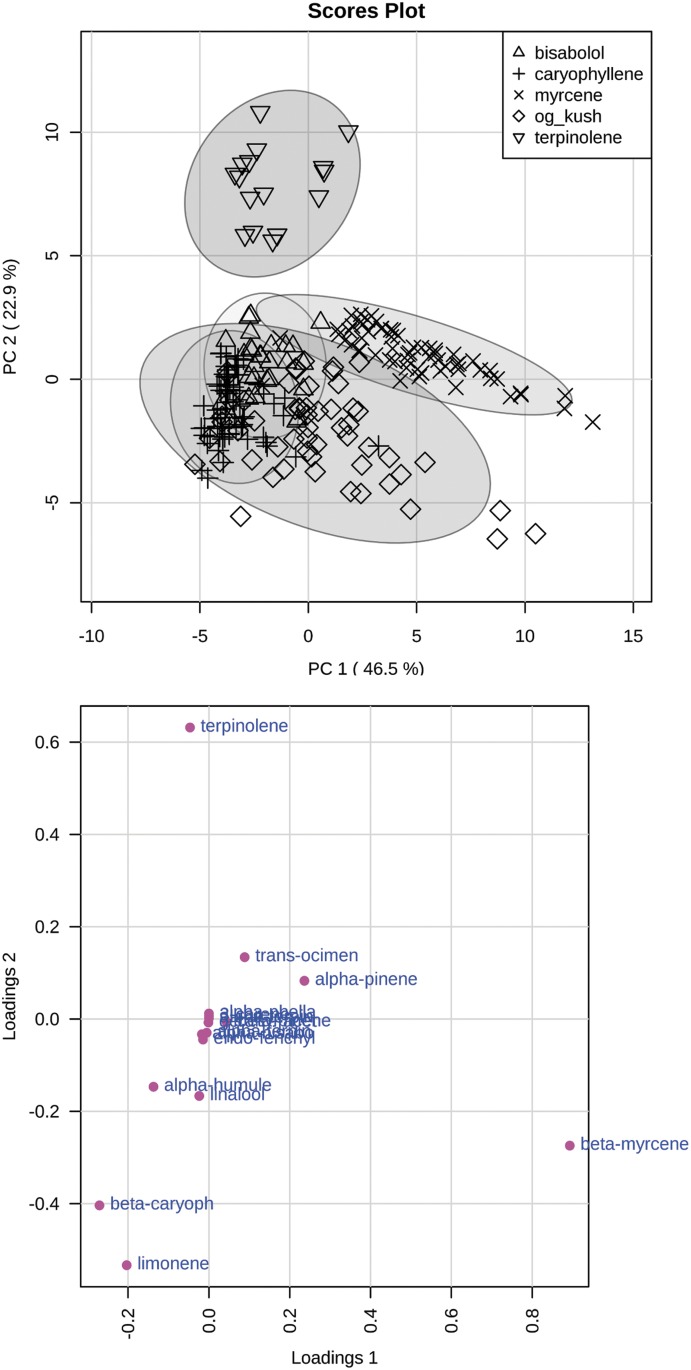
PCA scatter plot (top) and loading plot (bottom) of 5 terpenoid groups terpinolene, myrcene, caryophyllene, bisabolol, and og kush. PCA, principal component analysis.

To explore the above categorization, data were submitted to PCA (Fig. 2). Figure 2 shows the scores plot of Principal component 1 (PC1) versus PC2 with samples labeled according to the five groups identified in the HCA. PC1 explained 46.5% of variance, and PC2 explained 22.9% of variance in the data (total 69.4%). If PC3 is included, 85% of variance is explained (Supplementary Fig. S2). The higher percentage of variance explained in our model, compared with previous studies,^29,30^ is most likely from avoiding unnecessary variance from cannabinoid content as well as reasonable quantitative consistency of the terpenoid levels of the samples tested. The terpinolene category clearly clustered by itself along the negative PC1 and positive PC2 axes. The myrcene category also clustered mainly by itself along the positive PC1 axis. The Og Kush group partially overlapped with the caryophyllene, and bisabolol group along the negative PC1 axis, whereas very little overlap was observed along the positive PC1 axis. The loadings plot confirmed that the distinguishing characteristics of the terpinolene and myrcene groups were their respective dominant terpenoids, whereas relatively higher levels of limonene, β-caryophyllene, humulene, and linalool were characteristics of the Og Kush, β-caryophyllene, and α-bisabolol groups.

To further discriminate cultivars within the major groups identified by HCA and PCA, metabolite patterns of the cultivars were analyzed with supervised multivariate data analysis techniques. PLS-DA is a multivariate regression method that can be used to evaluate the differences between defined classes in a data matrix. OPLS-DA is an extension of PLS-DA that employs orthogonal signal correction, and was used here to identify variables (terpenoids) important for discriminating the cultivar classes. In OPLS-DA, the *X*-axis displays the maximum variation between classes, whereas the *Y*-axis displays maximum variation within classes.^42^

**FIG. 3.**
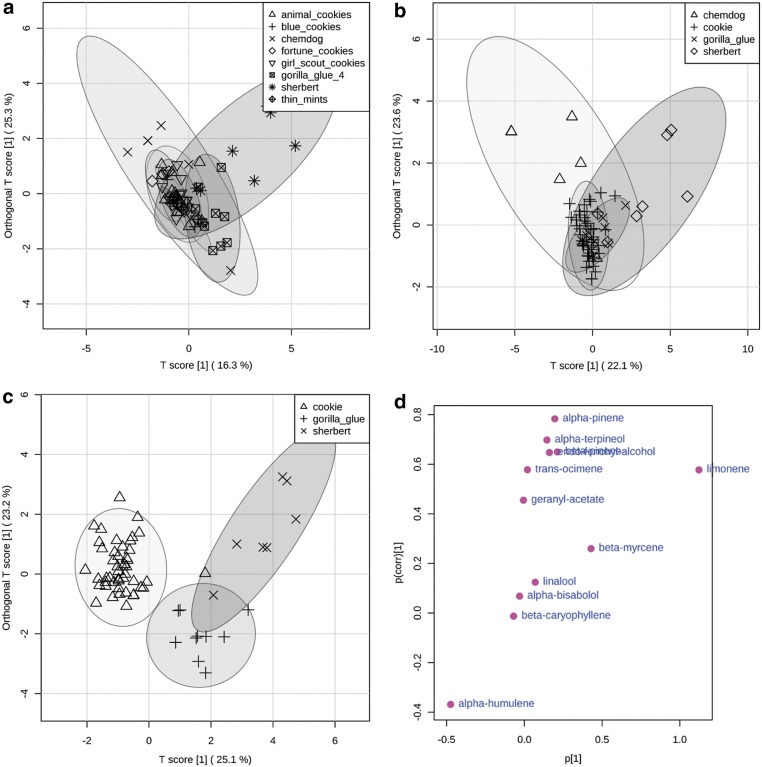
OPLS-DA of caryophyllene group. Score plot of cultivar name as classes **(a)**. Score plot of Chemdog, Cookie, Gorilla Glue, and Sherbert as classes **(b)**. Score plot of Cookie, Gorilla Glue, and Sherbert as classes **(c)**. S-plot of Cookie, Gorilla Glue, and Sherbert classes **(d)**. OPLS-DA, orthogonal partial least squares discriminant analysis.

The approach taken with OPLS-DA to aid in explaining the differences between cultivars within their respective groups is illustrated in detail with the β-caryophyllene group (Fig. 3). If all cultivars are assigned as their own class, clustering is observed among Blue Cookies, Girl Scout Cookies, Animal Cookies, Thin Mints, and Fortune Cookies, whereas Gorilla Glue #4 and Sherbert clustered more to themselves. Chemdog samples displayed a large within-class variation (Fig. 3a). Inspection of the quantitative data reveals that Blue Cookies, Girl Scout Cookies, Animal Cookies, Thin Mints, and Fortune Cookies shared the characteristic of the next most abundant terpenoids after β-caryophyllene; namely limonene and humulene in a ∼1:1 ratio. These cultivars were thus assigned to a class termed “Cookie” due to their similarity in nomenclature. Gorilla glue contrasted with the cookie cultivars in that limonene and myrcene (∼1:1) were the next most abundant terpenoids after β-caryophyllene. Sherbert contained the highest levels of limonene and higher levels of the alcohol-substituted monoterpenoid, linalool, followed by endo-fenchyl-alcohol, and α-terpineol.

When analyzed again, with cookie as a defined class, better clustering was observed within the cookie class, although it was still difficult to observe the distinction between Gorilla Glue, Sherbert, and Chemdog (Fig. 3b). The larger within-class variation of the Chemdog samples is most likely a result of the high variation in myrcene levels. Chemdog was removed from the data set and the remaining samples were analyzed again by OPLS-DA. Distinct clustering was thus observed between the Cookie, Gorilla Glue, and Sherbert classes (Fig. 3c). The S-plot shows significant metabolites contributing to the variation across the defined classes (Fig. 3d). Metabolites in the upper right and lower left corners of the S-plot (i.e., with the highest positive and lowest negative *p*-value versus *p*[corr]-values) were the most significant variables responsible for the discrimination between classes. The S-plot confirmed that the main terpenoids distinguishing the classes within the β-caryophyllene group are relatively higher levels of humulene in the cookies category, relatively higher levels of limonene and alcohol substituted terpenoids in the sherbert class, and relatively higher levels of myrcene in the Gorilla Glue class.

**FIG. 4.**
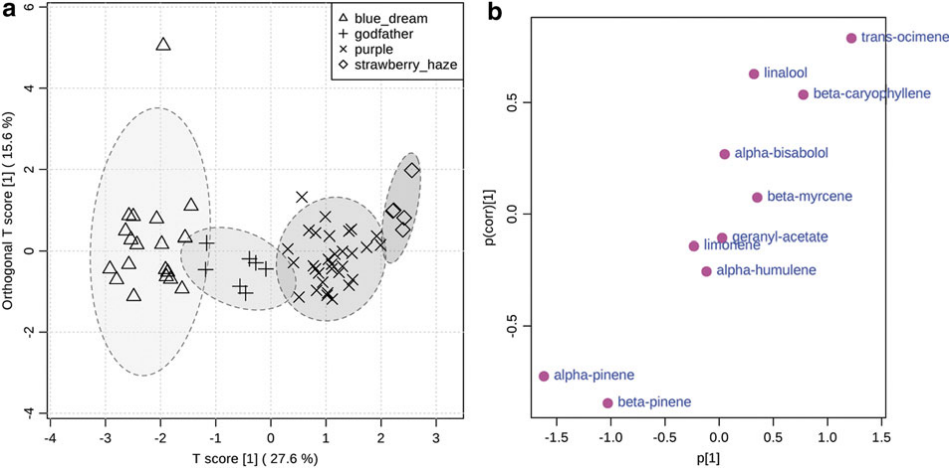
OPLS-DA of myrcene group with Blue Dream, Godfather, Purple, and Strawberry Haze as classes **(a)**. S plot of myrcene group **(b)**.

Inspection of the quantitative data within the myrcene group suggested that four classes could potentially be distinguished. Blue dream contained higher levels of α-pinene and β-pinene compared with all other cultivars in this study. The ratio between α-pinene, β-pinene, and myrcene was consistent at ∼2:1:4 throughout the samples of Blue Dream analyzed. Godfather contained on average the highest levels of myrcene followed by α-pinene and limonene. Godfather also contained lower amounts of β-caryophyllene and humulene relative to other cultivars in the myrcene group. Cultivars named Purple Cream, Grape Ape, Purple Princess, and Purple Urkle dominated in myrcene followed by α-pinene in a ∼4:1 ratio followed by *trans*-ocimene. Therefore, Purple Cream, Grape Ape, Purple Princess, and Purple Urkle were assigned to a class termed “Purple” due to their shared morphological characteristic of purple-tinged flower buds. Strawberry Haze had the highest levels of *trans*-ocimene followed by limonene. When analyzed by OPLS-DA, the samples in each of the assigned classes within the myrcene group cluster, and clusters are separated along the first principal component (Fig. 4a). The S-plot confirmed that *trans*-ocimene and linalool were the most important distinguishing terpenoids for the Strawberry Haze and Purple categories, whereas α-pinene and β-pinene were the most important for distinguishing the Blue Dream category (Fig. 4b).

**FIG. 5.**
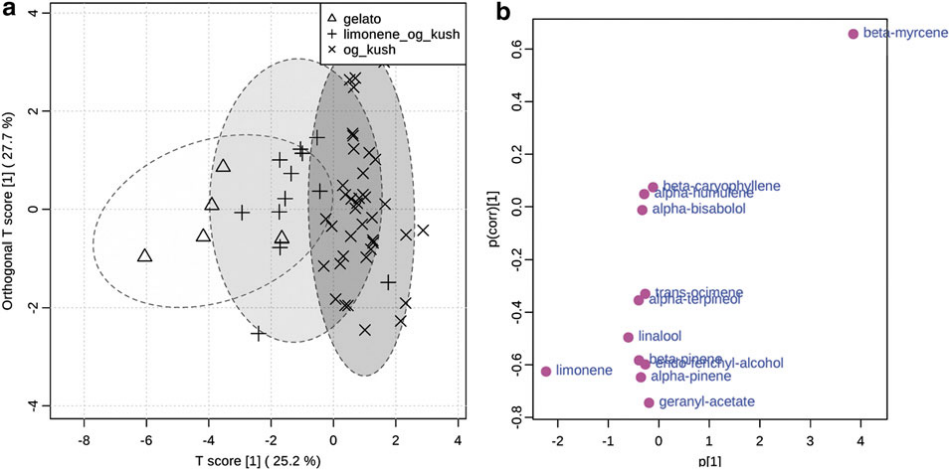
OPLS-DA of Og Kush group with Gelato, Limonene Og Kush, and Og Kush as classes **(a)**. S plot of Og Kush group **(b)**.

Within the Og Kush group, Crown Og, Gas, Og Kush, Skywalker Og Kush, Superman Og Kush, and Tahoe Og Kush cultivars all tended to dominate in myrcene with similar or slightly lower levels of limonene. These cultivars were assigned to a class termed “Og Kush” due to similarity in nomenclature. Miami White Kush and Triple O dominated in limonene compared with myrcene in a ∼2:1 ratio and were assigned to a class termed “Limonene Og Kush.” Gelato dominated in limonene, but contained even lower amounts of myrcene. Gelato also tended to contain the highest levels of linalool, endo-fenchyl-alcohol, α-terpineol, and geranyl acetate compared with other cultivars. OPLS-DA analysis of these classes revealed a continuum of terpenoid profiles with some overlap between Og Kush and Limonene Og Kush, as well as between Limonene Og Kush and Gelato (Fig. 5a). The S-plot confirmed that the main source of variation within the Og Kush group is whether the chemotype tends to dominate in myrcene or limonene (Fig. 5b).

Jack Herer and Trainwreck cultivars were distinguishable in a previous study^29^ and bear strong resemblance to the Bedrocan cultivar available through Dutch pharmacies as well as other accessions described as sativa dominant.^28,39^ Within the terpinolene group, Jack Herer mainly differed from Trainwreck due to lower levels of myrcene and limonene. Another important characteristic of the terpinolene chemotype is the presence of α-phellandrene and 3-carene. As discussed in Hazekamp 2016,^30^ β-phellandrene overlaps with limonene. GC-MS analysis of representative terpinolene chemotype samples revealed that the peak at RRT 0.377 is most likely composed of β-phellandrene and limonene. In representative samples of cultivars that lack α-phellandrene and terpinolene, the limonene peak does not seem to contain β-phellandrene (Supplementary Fig. S3). Although the quantitative analysis failed to detect 3-carene and α-terpinene above detection limits in Jack Herer samples by GC-FID, the presence of these compounds was confirmed by GC-MS analysis of a representative Jack Herer sample (Supplementary Fig. S1B). Since the terpinolene chemotype had such a distinctive profile no further analysis was necessary.

**FIG. 6.**
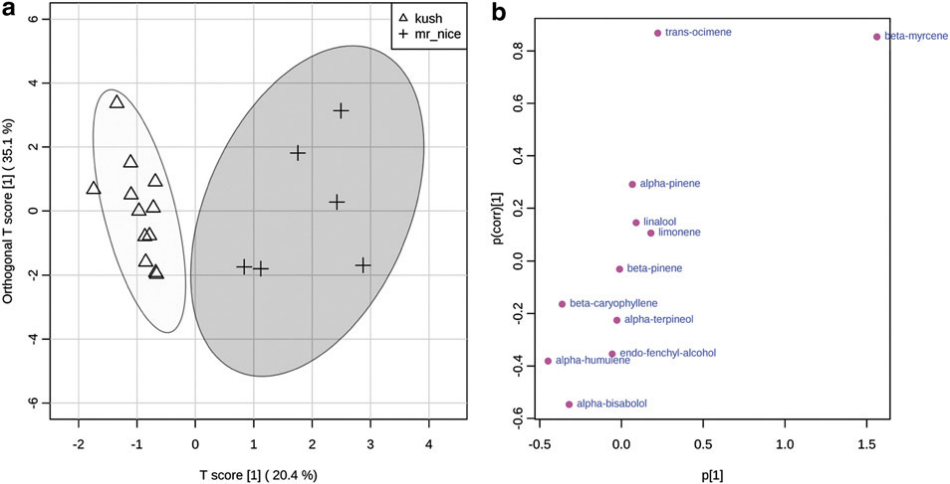
OPLS-DA of bisabolol group with Kush and Mr. Nice as classes **(a)**. S plot of Kush and Mr. Nice classes **(b)**.

In the bisabolol group Master and Bubba Kush tended to dominate in limonene, β-caryophyllene, and myrcene in a ∼2:2:1 ratio. Mr. Nice had a ∼1:1:1 ratio of myrcene, limonene, and β-caryophyllene. Initial OPLS-DA analysis with each cultivar as class resulted in poor clustering for Sour Diesel likely due to high variation in the sample set and lack of other distinguishing characteristics (Supplementary Fig. S4). If Bubba Kush and Master Kush were combined into a class termed “Kush” and analyzed by OPLS-DA with Mr. Nice discrimination is observed (Fig. 6a). The S-plot confirmed that Mr. Nice was distinguishable due to its higher levels of myrcene, whereas the Kush class had more humulene and alcohol-substituted terpenoids, mainly α-bisabolol (Fig. 6b).

Based on the overall terpenoid profiles, HCA, PCA, and OPLS-DA, a scheme for classifying the cultivars was developed based on dominant terpenoids, ratios of major terpenoids, presence of unique terpenoids, or other characteristic terpenoids (Table 3). To test this classification approach, different PLS-DA models were analyzed with different sample sets and class assignments (Table 4). Sour Diesel and Chemdog samples were not included in PLS-DA models. In model 1, cultivar names were assigned as classes. In model 2, class names from Table 3 were used as classes. In model 3, samples from the data collection period that contained three to four replicates and could be assigned to the classes in Table 3 were added (Table 3 italics). This included an additional 57 samples representing 17 additional cultivar names (Supplementary Table S1). In model 4, only classes that contained >15 samples (from the sample set in model 3) per class were used. This included the Blue Dream, Cookie, Og Kush, Limonene Og Kush, Purple, and Terpinolene classes.

**Table 3.** **Classification Table of Major Group Terpenoid Groups and Cultivars Assigned to Class Names**

Major group characteristics	Secondary characteristics	Class name	Cultivars names
Terpinolene dominant	α-Phellandrene, β-phellandrene 3-carene, α-terpinene	Terpinolene	Trainwreck, Jack Herer, *Ace of Spades, Sage*
β-Caryophyllene-dominant, alcohol-substituted terpenoids	Limonene: humulene ∼1:1	Cookie	Animal Cookies, Blue Cookies, Fortune Cookies, Girl Scout Cookies, Thin Mints, *Cookie, Phantom Cookies*
	Limonene: humulene ∼2:1	Sherbert	Sherbert, *Sunset Sherbert*
	Limonene: myrcene ∼1:1	Glue	Gorilla Glue #4, *Super Glue*
Limonene/myrcene-dominant, alcohol-substituted terpenoids	Limonene: myrcene ∼1:1	Og Kush	Crown Og, Gas, Og Kush, Skywalker Og Kush, Superman Og Kush, Tahoe Og Kush, *Hardcore Og, Louis XIII Og Kush, Milky Way Og Kush, Wifi Og Kush*
	Limonene: myrcene ∼2:1	Limonene Og Kush	Miami White Kush, Triple O, *Headband*
	Limonene: linalool ∼3:1	Gelato	Gelato
Limonene/myrcene/β-caryophyllene dominant, α-bisabolol	Limonene: β-caryophyllene: myrcene ∼2:2:1	Kush	Bubba Kush, Master Kush
	Limonene: myrcene: β-caryophyllene ∼1:1:1	Mr. Nice	Mr. Nice
Myrcene dominant	α-Pinene >*trans*-ocimene	Purple	Grape Ape, Purple Cream, Purple Princess, Purple Urkle, *Blue Mazaar, Granddaddy Purple, Purple Max, Watermelon*
	α-Pinene: β-pinene ∼2:1	Blue Dream	Blue Dream
	*trans*-Ocimene >limonene	Strawberry	Strawberry Haze, *Strawberry Cough*
	α-Pinene >limonene	High myrcene	Godfather, *AK-47*

Cultivars in italics were added to PLS-DA model 3.

PLS-DA, partial least squares discriminant analysis.

**Table 4.** **Partial Least Squares Discriminant Analysis Model Classification Inputs and *Q*^2^ (Cross-Validated *R*^2^) Value from Five Component Model**

PLS-DA class assignments	Model number	Samples in model	Classes in model	*Q*^2^
Cultivar name as classes	1	217	30	0.29750
Class names from Table 3 classes	2	217	13	0.54896
Cultivars with three to four samples added to model 2	3	274	13	0.57430
Classes with >15 samples	4	208	6	0.81654

The PLS-DA models were subjected to cross-validation and permutation tests.^43^ For cross-validation five components were searched and leave-one-out cross-validation was used. Prediction accuracy, *R*^2^ (sum of squares captured by model) and *Q*^2^ (cross-validated *R*^2^) were plotted and *Q*^2^ used as performance measure. The closer *Q*^2^ is to 1, the better the goodness of prediction of the PLS-DA model. The best performance of the models (as indicated by the red asterisk in the plots) was achieved using all five components (Supplementary Figs. S5–S8, top). From models 1 to 2, *Q*^2^ increased substantially with only minor increase in *Q*^2^ between models 2 and 3, whereas model 4 has the highest *Q*^2^ value (Table 4). Permutation tests were performed on each model to determine whether discrimination between classes were statistically significant, or due to random noise.^43^ Two thousand permutations were performed, during each of which a five-component PLS-DA model was built between the terpenoid data and permuted class labels. Histograms show permutation test scores, and compare it to the performance based on the defined classifications (red arrow) (Supplementary Figs. S5–S8, bottom). The further the separation distance, based on a sum of squares between/sum of squares (B/W) ratio, between the observed statistic and the distribution resulting from permuted data, the more significant the discrimination.^44^ The separation difference increased from model 1 to 2 from 2 to 3 and from 3 to 4 (Supplementary Figs. S5–S8, bottom). The PLS-DA results confirm that classification based on terpenoid classes outperforms classification based on cultivar name. When new samples were added to the sample set from models 1 and 2, a slightly improved model 3 resulted. However, the highest performing model was achieved with classes that contain ≥15 samples (model 4). These results suggest that better predictive models are constructed from terpenoid profiles with more representative samples.

## Conclusion

This study has demonstrated an approach to discriminating terpenoid chemotypes among cannabis cultivars, despite obscure nomenclature. Overall, a hierarchy of chemotype was observed that could initially be broken down into five major terpenoid groups based on dominant terpenoid and relative levels of hydroxylated terpenoids. These five major groups could be broken down into 13 classes. The Cookie, Og Kush/Limonene Og Kush, Purple, and Terpinolene classes were clearly distinguishable chemotypes comprised of many representative cultivar names. Blue Dream represented a chemotype with only one cultivar name. The remaining classes could represent either new chemotypes pending confirmation from more representative samples, or rather a continuum of variation within a larger chemotype. More sensitive methods for terpenoid analysis in cannabis samples such as a recently published method by Giese et al.^45^ as well as the unequivocal identification of difficult-to-resolve sesquiterpenoids in cannabis would aid classification efforts. Information about terpenoid chemotypes can allow doctors and clinical researchers to design studies to assess whether they have different medicinal or subjective effects, despite similar cannabinoid content. Since it is unlikely that the popularly used cultivar names (“strain” names as they are commonly referred to in the cannabis industry) will go away, the chemotype approach allows a more objective way of understanding cannabis chemical diversity for the newly emerging cannabis industry. Combining chemotaxonomic data, with morphological and genetic data, would provide a more complete picture of cannabis taxonomy.

## Supplementary Material

Supplemental data

Supplemental data

Supplemental data

Supplemental data

Supplemental data

Supplemental data

## Abbreviations Used

CBC: cannabichromene
CBD: cannabidiol
CBG: cannabigerol
CBN: Cannabinol
EU: European Union
FID: flame ionization detector
GC-MS: gas chromatography–mass spectrometry
HCA: hierarchical clustering
MeOH: methanol
OPLS-DA: orthogonal partial least squares discriminant analysis
PC1: Principal component 1
PCA: principal component analysis
PLS-DA: partial least squares discriminant analysis
THC: tetrahydrocannabinol
THCV: tetrahydrocannabivarin
RRT: relative retention time
