# Supplementary material for: Identification of Terpenoid Chemotypes Among High (−)-trans-Δ9- Tetrahydrocannabinol-Producing Cannabis sativa L. Cultivars
Source: Cannabis Cannabinoid Res. 2017 Mar 1;2(1):34–47. doi: 10.1089/can.2016.0040 (PMC5436332; doi:10.1089/can.2016.0040)
Supplement: Supplemental data [file Supp_Fig1.pdf]

Supplementary Data

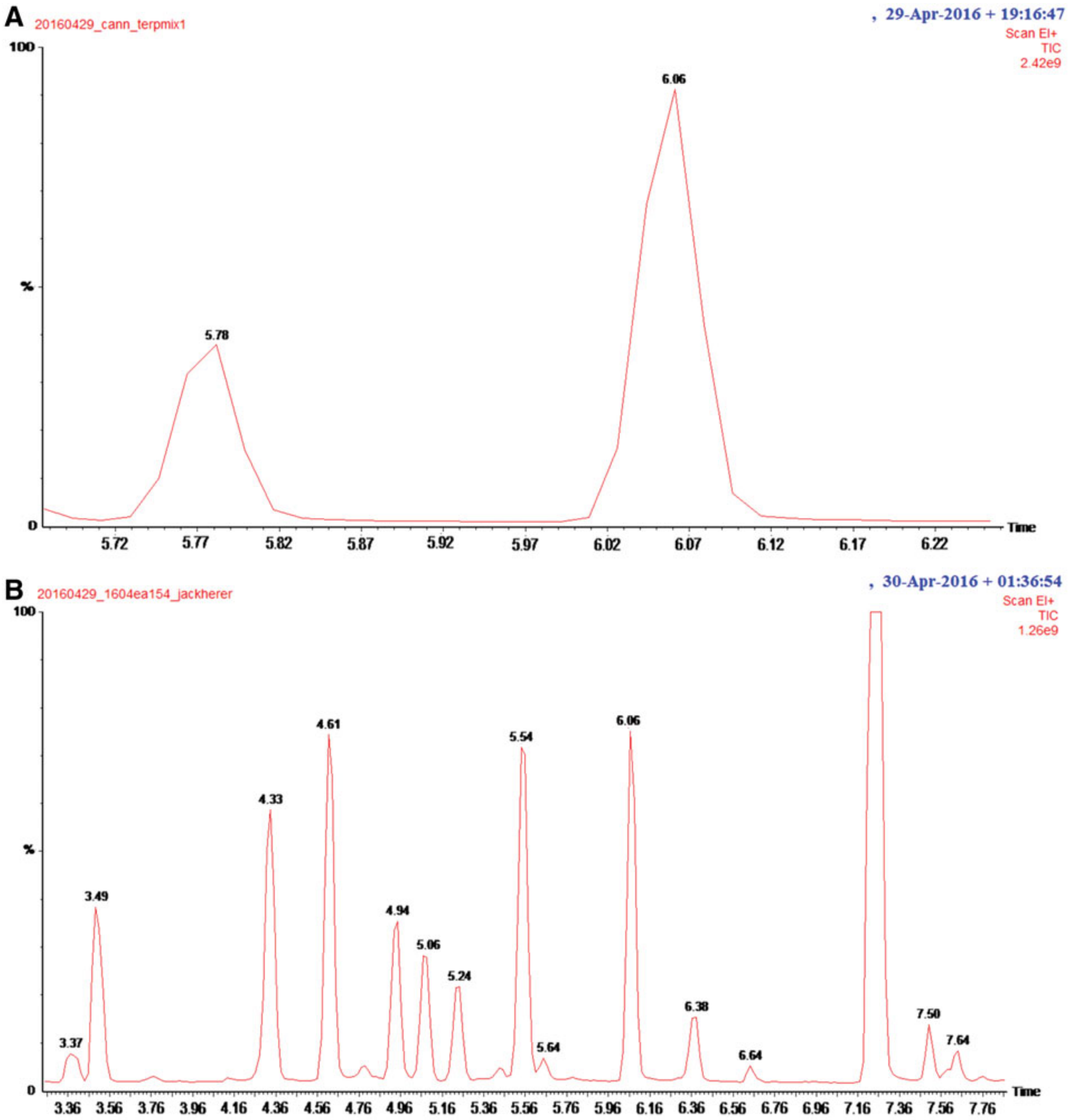

SUPPLEMENTARY FIG. S1. GC-MS chromatograms and mass spectra of *cis*-ocimene and *trans*-ocimene in reference mix and representative Jack Herer sample. **(A)** Ocimene peaks in CannTerpMix 1 (*cis*-ocimene 5.78 min, *trans*-ocimene 6.06 min). **(B)** Representative Jack Herer sample with α-phellandrene 4.94 min, 3-carene 5.06 min, α-terpinene, 5.24 min, and *trans*-ocimene 6.06 min. **(C)** Mass spectrum of *cis*-ocimene standard. **(D)** Mass spectrum of *trans*-ocimene standard. **(E)** Mass spectrum of *trans*-ocimene in Jack Herer sample. GC-MS, gas chromatography–mass spectrometry.

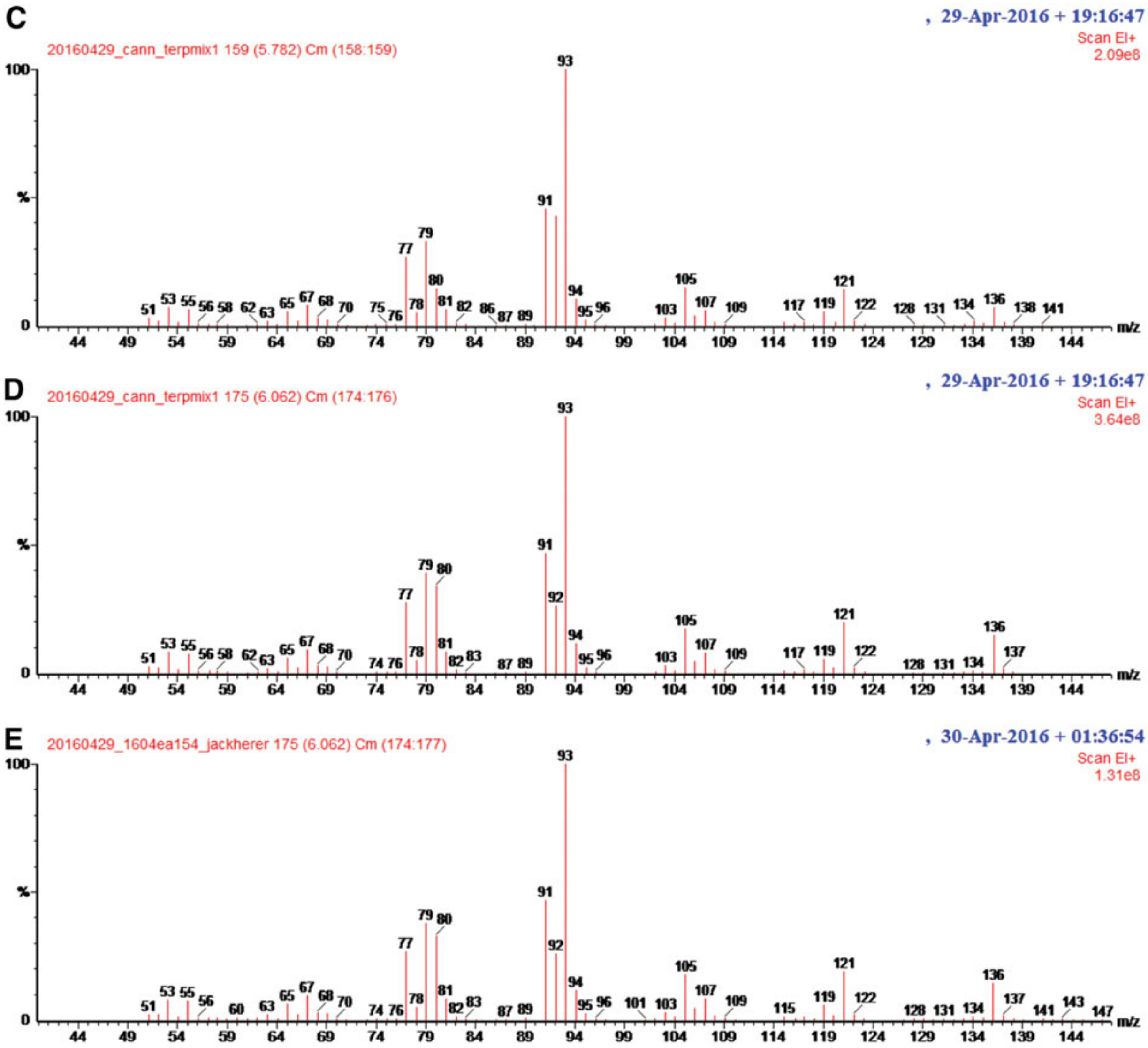

SUPPLEMENTARY FIG. S1. (Continued).
