# Supplementary material for: Identification of Terpenoid Chemotypes Among High (−)-trans-Δ9- Tetrahydrocannabinol-Producing Cannabis sativa L. Cultivars
Source: Cannabis Cannabinoid Res. 2017 Mar 1;2(1):34–47. doi: 10.1089/can.2016.0040 (PMC5436332; doi:10.1089/can.2016.0040)
Supplement: Supplemental data [file Supp_Fig2.pdf]

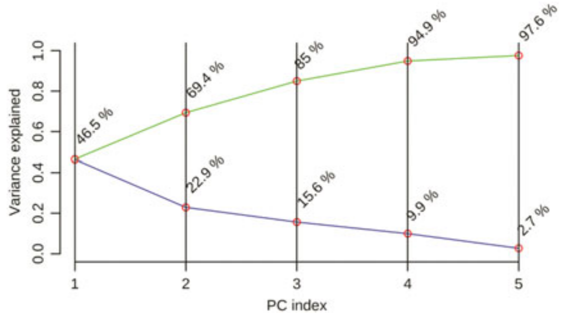

SUPPLEMENTARY FIG. S2. Principal component total variance explained (green line) and individual component variation explained (blue line).
