# Supplementary material for: Identification of Terpenoid Chemotypes Among High (−)-trans-Δ9- Tetrahydrocannabinol-Producing Cannabis sativa L. Cultivars
Source: Cannabis Cannabinoid Res. 2017 Mar 1;2(1):34–47. doi: 10.1089/can.2016.0040 (PMC5436332; doi:10.1089/can.2016.0040)
Supplement: Supplemental data [file Supp_Fig3.pdf]

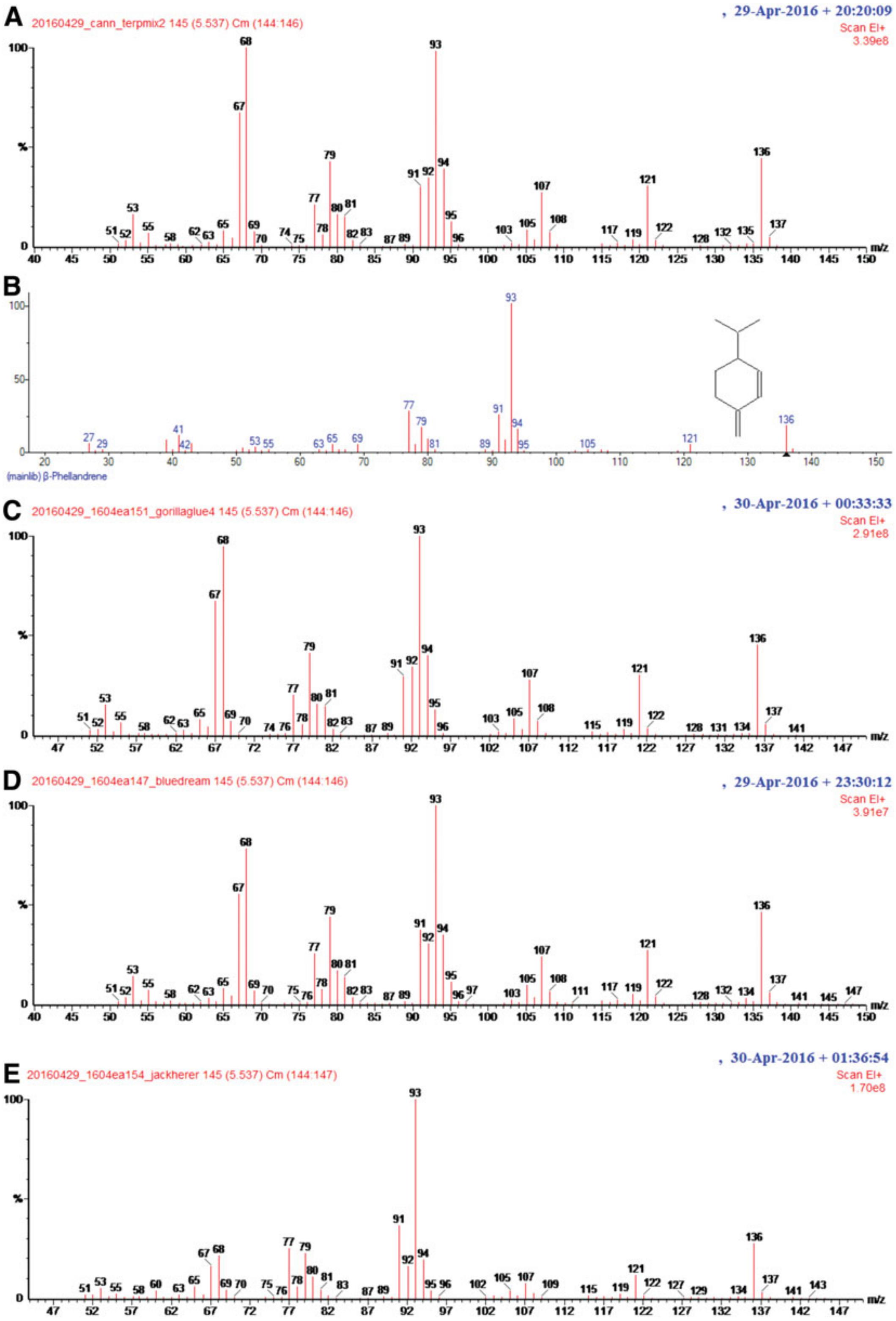

SUPPLEMENTARY FIG. S3. **(A)** Mass spectrum of limonene in CannTerpMix 2. **(B)** Mass spectrum of β-phellandrene in NIST library Version 2.0. **(C)** Limonene peak in a sample of Gorilla Glue #4. **(D)** Limonene peak in a sample of Blue Dream. **(E)** Peak containing β-phellandrene overlapping with limonene in Jack Herer sample.
