# Supplementary material for: Identification of Terpenoid Chemotypes Among High (−)-trans-Δ9- Tetrahydrocannabinol-Producing Cannabis sativa L. Cultivars
Source: Cannabis Cannabinoid Res. 2017 Mar 1;2(1):34–47. doi: 10.1089/can.2016.0040 (PMC5436332; doi:10.1089/can.2016.0040)
Supplement: Supplemental data [file Supp_Fig4.pdf]

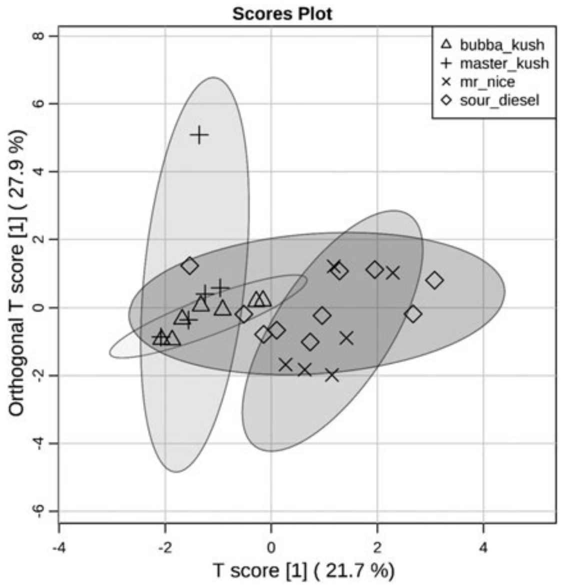

SUPPLEMENTARY FIG. S4. OPLS-DA analysis of bisabolol group with cultivar names as classes. OPLS-DA, orthogonal partial least squares discriminant analysis.
