# Supplementary material for: Identification of Terpenoid Chemotypes Among High (−)-trans-Δ9- Tetrahydrocannabinol-Producing Cannabis sativa L. Cultivars
Source: Cannabis Cannabinoid Res. 2017 Mar 1;2(1):34–47. doi: 10.1089/can.2016.0040 (PMC5436332; doi:10.1089/can.2016.0040)
Supplement: Supplemental data [file Supp_Table1.pdf]

Supplementary Table S1. Quantitative Terpenoid Data (mg/g) of Cultivars Added to Partial Least Squares Discriminant Analysis Model 3

Cultivar name	Sample (n=#)	α-Pinene	β-Pinene	Myrcene	α-Phellandrene	3-Carene	α-Terpinene	Limonene[a]	trans-Ocimene	Terpinolene	Linalool	Endo-fenchyl-alcohol	α-Terpineol	Geranyl-acetate	β-Caryophyllene	α-Humulene	α-Bisabolol
Ace of Spades	3	1.2 ± 0.1	1.8 ± 0.3	5.4 ± 0.3	0.7 ± 0.1	0.6 ± 0.1	< LOQ	5.4 ± 0.8	3.3 ± 0.7	13.9 ± 1.9	0.5 ± 0.1	0.5 ± 0.1	0.7 ± 0.1	0.4 ± 0.4	1.2 ± 0.1	0.5 ± 0.1	ND
Ak 47	3	1.8 ± 0.3	0.9 ± 0.3	11.8 ± 6.4	ND	ND	ND	1.3 ± 0.2	0.6 ± 0.5	ND	ND	ND	ND	ND	3.7 ± 2.7	1.6 ± 1.0	0.9 ± 1.0
Blue Mazar	4	1.6 ± 0.2	< LOQ	6.7 ± 0.6	ND	ND	ND	ND	1.2 ± 0.1	ND	1.2 ± 0.3	ND	ND	ND	4.1 ± 0.9	1.1 ± 0.3	< LOQ
Cookies	3	ND	0.6 ± 0.1	1.9 ± 0.7	ND	ND	ND	4.0 ± 0.6	ND	ND	1.9 ± 0.7	< LOQ	< LOQ	ND	6.6 ± 1.1	3.4 ± 0.5	0.4 ± 0.3
Granddaddy Purple	4	2.3 ± 0.6	0.6 ± 0.1	6.7 ± 2.8	ND	ND	ND	ND	1.0 ± 0.5	ND	1.2 ± 0.2	ND	ND	ND	3.6 ± 0.7	1.0 ± 0.2	< LOQ
Hardcore Og	3	0.4 ± 0.4	1.2 ± 0.3	6.1 ± 1.1	ND	ND	ND	5.9 ± 1.9	ND	ND	2.1 ± 0.7	0.7 ± 0.2	0.7 ± 0.2	ND	3.3 ± 1.3	1.3 ± 0.4	< LOQ
Headband	3	< LOQ	0.9 ± 0.2	1.9 ± 0.8	ND	ND	ND	5.7 ± 1.3	ND	ND	1.2 ± 0.0	0.6 ± 0.1	ND	ND	4.5 ± 0.9	1.8 ± 0.3	0.9 ± 0.2
Louis XIII Og Kush	3	< LOQ	1.0 ± 0.3	6.7 ± 1.2	ND	ND	ND	5.5 ± 1.8	ND	ND	1.7 ± 0.8	0.5 ± 0.4	0.5 ± 0.4	ND	2.9 ± 0.3	1.1 ± 0.0	< LOQ
Milky Way Og Kush	4	< LOQ	0.9 ± 0.0	6.4 ± 1.8	ND	ND	ND	5.1 ± 0.2	ND	ND	1.7 ± 0.1	0.5 ± 0	0.5 ± 0	ND	3.4 ± 0.9	1.3 ± 0.3	< LOQ
Phantom Cookies	3	ND	< LOQ	2.5 ± 0.2	ND	ND	ND	3.2 ± 1.0	ND	ND	1.1 ± 0.5	< LOQ	ND	ND	6.1 ± 1.0	3.0 ± 0.5	< LOQ
Purple Max	4	2.1 ± 0.4	0.6 ± 0.1	8.1 ± 1.5	ND	ND	ND	ND	1.4 ± 0.2	ND	1.1 ± 0.1	ND	ND	ND	3.5 ± 0.4	0.9 ± 0.1	< LOQ
Sage	3	0.4 ± 0.3	0.9 ± 0.1	3.0 ± 1.6	ND	ND	ND	2.8 ± 0.4	3.0 ± 1.1	7.7 ± 1.2	< LOQ	ND	< LOQ	ND	3.6 ± 1.5	1.4 ± 0.6	0.4 ± 0.3
Strawberry Cough	4	1.0 ± 0.2	0.7 ± 0.2	7.0 ± 2.2	ND	ND	ND	2.4 ± 1.1	3.6 ± 0.9	ND	1.3 ± 0.3	ND	ND	ND	2.9 ± 1.1	1.4 ± 0.5	0.7 ± 0.2
Sunset Sherbert	3	0.9 ± 0.2	1.2 ± 0.2	1.9 ± 0.5	ND	ND	ND	5.5 ± 1.7	0.6 ± 0.2	ND	1.8 ± 0.2	0.7 ± 0.1	0.7 ± 0.1	0.7 ± 0.2	5.3 ± 0.5	2.3 ± 0.1	ND
Super Glue	4	ND	< LOQ	3.8 ± 0.1	ND	ND	ND	2.8 ± 0.7	ND	ND	< LOQ	< LOQ	ND	ND	7.7 ± 0.5	2.4 ± 0.1	0.9 ± 0.0
Watermelon	3	2.5 ± 0.8	0.7 ± 0.2	8.6 ± 1.9	ND	ND	ND	< LOQ	1.7 ± 0.3	ND	1.1 ± 0.1	ND	ND	ND	4.9 ± 1.5	1.3 ± 0.4	0.3 ± 0.3
Wifi Og Kush	3	0.4 ± 0.4	1.0 ± 0.4	3.1 ± 0.8	ND	ND	ND	5.1 ± 2.1	ND	ND	1.4 ± 0.3	0.5 ± 0.4	0.4 ± 0.4	ND	2.8 ± 0.4	1.0 ± 0.2	ND

[a]Limonene peak overlaps with β-phellandrene mainly in cultivars containing α-phellandrene.
Plus or minus values indicate standard deviation.
< LOQ, less than limit of quantification (<0.4 mg/g); ND, not detected.
