# Supplementary material for: Identification of Terpenoid Chemotypes Among High (−)-trans-Δ9- Tetrahydrocannabinol-Producing Cannabis sativa L. Cultivars
Source: Cannabis Cannabinoid Res. 2017 Mar 1;2(1):34–47. doi: 10.1089/can.2016.0040 (PMC5436332; doi:10.1089/can.2016.0040)
Supplement: Supplemental data [file Supp_Fig5-8.pdf]

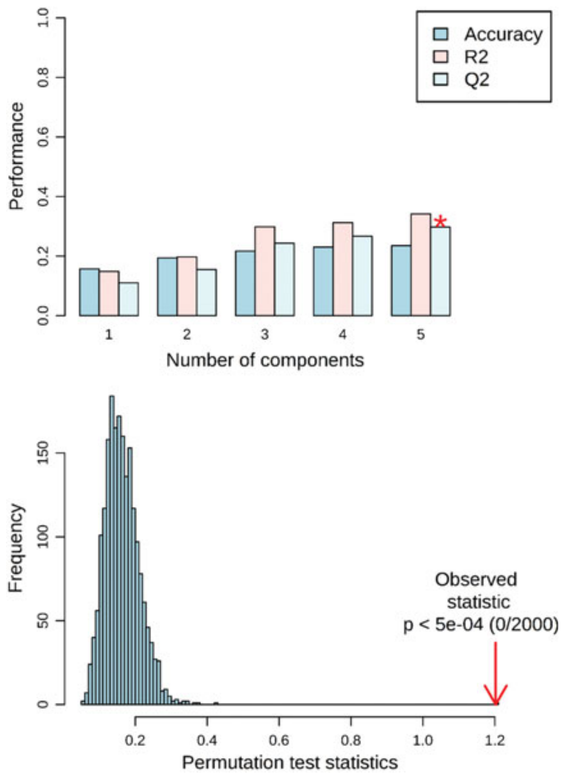

SUPPLEMENTARY FIG. S5. PLS-DA cross-validation results with cultivar names as classes (top). Permutation test results with cultivar names as classes (bottom). *Best performance of model. PLS-DA, partial least squares discriminant analysis.

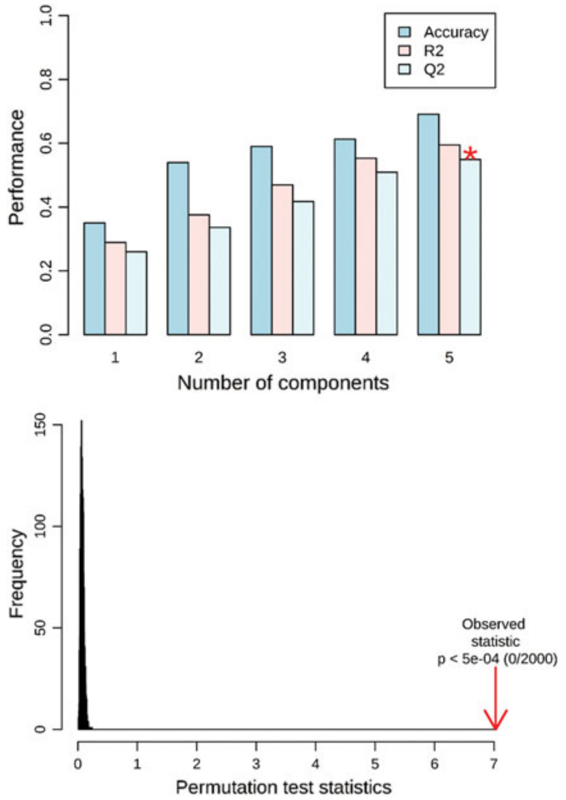

SUPPLEMENTARY FIG. S6. PLS-DA cross-validation results with class names as classes (top). Permutation test results with class names as classes (bottom). *Best performance of model.

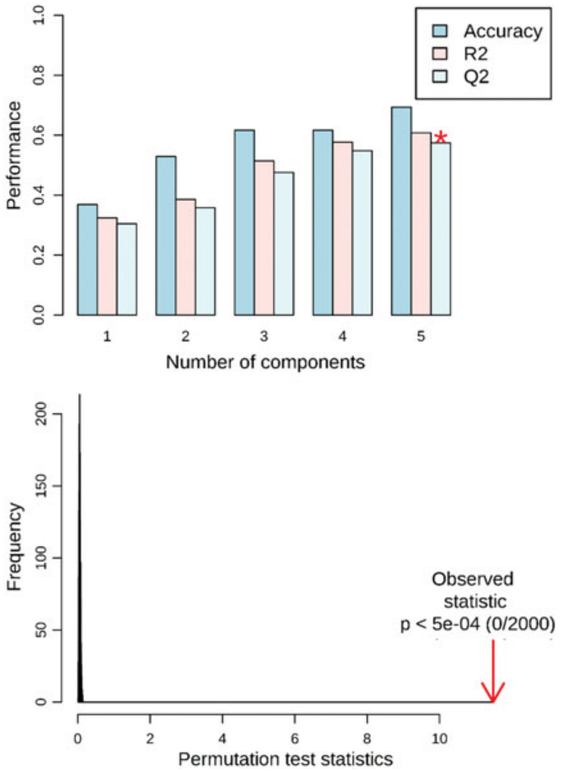

SUPPLEMENTARY FIG. S7. PLS-DA cross-validation results with new samples added to classes (top). Permutation test results with new samples added to classes (bottom). *Best performance of model.

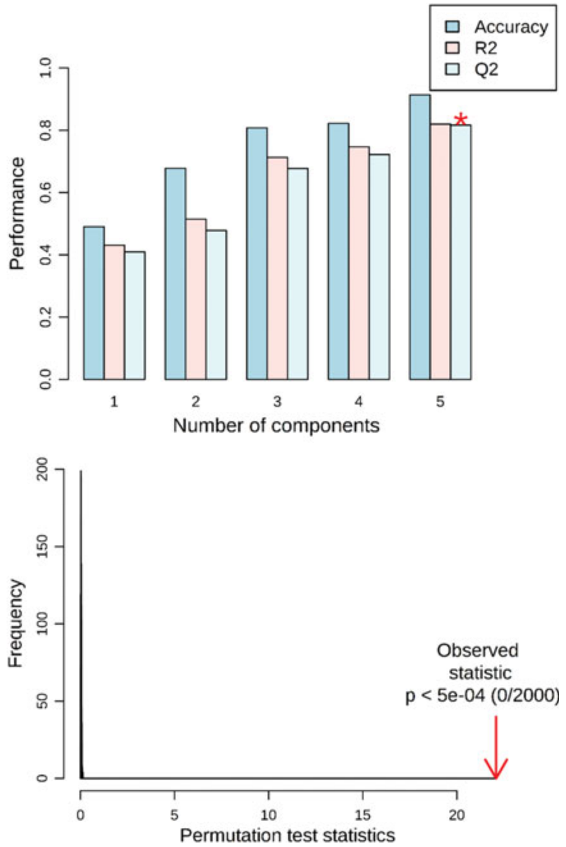

SUPPLEMENTARY FIG. S8. PLS-DA cross-validation results with classes containing $n \geq 15$ samples (top). Permutation test results with classes containing $n \geq 15$ samples (bottom). *Best performance of model.
